# A Hybrid Optical Fiber Detector for the Simultaneous Measurement of Dust Concentration and Temperature

**DOI:** 10.3390/s25144333

**Published:** 2025-07-11

**Authors:** Chuanwei Zhai, Li Xiong

**Affiliations:** 1 China Academy of Safety Science and Technology, Beijing 100012, China; 18266085850@163.com; 2 Key Laboratory of Metallurgical Equipment and Control Technology, Ministry of Education, Wuhan University of Science and Technology, Wuhan 430081, China; 3 Hubei Key Laboratory of Mechanical Transmission and Manufacturing Engineering, Wuhan University of Science and Technology, Wuhan 430081, China

**Keywords:** optical fiber detector, fiber Bragg grating (FBG), optical fiber collimator, dust concentration measurement, temperature measurement

## Abstract

This work presents a hybrid optical fiber detector by combining the sensing mechanism of the fiber Bragg grating (FBG) and the light extinction method to enable the simultaneous measurement of dust concentration and temperature. Compared with the existing dust concentration sensors, the proposed detector offers three key advantages: intrinsic safety, dual-parameter measurement capability, and potentially network-based monitoring. The critical sensing components of the proposed detector consist of two optical collimators and an FBG. Using the extinction effect of light between the two collimators, the dust concentration and temperature are simultaneously determined by monitoring the intensity and the wavelength of the FBG reflectance spectrum, respectively. The measurement feasibility has been evaluated demonstrating that the two parameters of interest can be effectively sensed with minimally coupled outputs of ±3 pm and ±0.1 mW, respectively. Calibration experiments demonstrate that the change in the intensity of light from the FBG is exponentially related to the dust concentration variation with fitting coefficients equal to 0.948, 0.946, and 0.945 for 200 meshes, 300 meshes, and 400 meshes, respectively. The detector’s relative measurement errors were validated against the weighing method, confirming low measurement deviations.

## 1. Introduction

Combustible dust has caused frequent explosion accidents, leading to human casualties, substantial economic losses, and significant damage in fields such as the chemical industry, metallurgy, food processing, and mineral mining, among others [1,2,3,4]. For example, in 2015, a dust explosion tragedy in New Taipei Amusement Park, Taiwan, caused 12 deaths and 498 injuries. Combustible dust explosions pose a serious threat to public safety and property [5].

Factors affecting the likelihood of a dust explosion are dust concentration, dust particle size, dust components, lower explosive limit (LEL), and minimum ignition energy (MIE). Among these, dust concentration is the most significant factor influencing explosions [6,7]. Meanwhile, ambient temperature is also an essential parameter affecting dust explosions. A higher temperature lowers the explosive limit and makes it easier to cause an explosion [8]. Effective monitoring methods can provide scientific data on dust concentration and temperature distribution, crucial for preventing combustible dust explosions.

Commonly used dust concentration measurement methods include capacitive [9], *β*-ray [10], light scattering [11,12], light extinction [13,14] and electrostatic induction [15,16] techniques. The capacitive method has a simple principle. However, the capacitive sensors are susceptible to noise, especially in mesh configurations, due to crosstalk, field interactions, and fringing capacitance. Moreover, capacitive sensors require relatively complex electronics to filter the noise, as mentioned earlier [17]. The *β*-ray method is an indirect technique with high accuracy. However, it requires the dust concentration to be measured after sampling, making real-time online monitoring challenging. Furthermore, the radioactivity of the *β*-ray source poses a safety risk.

Currently, several commercial products have been developed based on electrostatic induction [18]. This technology for dust concentration detection offers the advantages of low maintenance, high sensitivity, low cost, and a simple structure. A charge imbalance occurs at a negatively charged electrode as charged dust particles pass it. Thus, dust concentration can be measured by analyzing charge fluctuations on the electrode surface. In the latest research, an internally inserted electrostatic sensor array composed of nine pairs of electrodes was used to measure the concentration distribution of the entire cross-section of the pipeline [19].

Light scattering and light extinction methods are also the mainstream dust concentration measurement techniques that have been successfully applied to monitor dust concentrations in flues and coal mines. The measurement principles of these two methods are similar. When dust particles are irradiated by LED-generated light, scattering and absorption occur due to interaction with the particles. Given specific optical system configurations and dust properties, the intensities of scattered and transmitted light are proportional to the dust concentration. These intensities are detected and converted by the phototransistor within the sensor.

These sensors offer certain benefits. However, they exhibit several limitations: first, the sensors mentioned above are all active devices requiring an electrical power supply at the dust monitoring site, which may pose a spark ignition risk from charge accumulation, short circuits, or circuit failures, thereby increasing the explosion risk. Second, in large-area monitoring sites, such as coal mines and pipelines, it is necessary to gain information about the spatial distribution of the dust concentration from multiple sensors located at different site locations. Such deployments are suited for point monitoring but make networked monitoring and data fusion challenging.

Fiber Bragg grating (FBG)-based sensing technology has gained significant importance for numerous applications due to its inherent advantages, such as immunity to electromagnetic interference and optical power fluctuations, small size, lightweight, and the capability for multiplexing multiple FBGs along a single fiber [20,21,22]. Leveraging these advantages, this research proposes an FBG-based dust detector that integrates FBG sensing with a modified light extinction method.

The innovations and contributions of this research can be extracted as follows: (1) The proposed detector has abandoned the standard optoelectronic components and instead utilizes fiber collimators and FBG as the primary sensing devices to achieve uncharged measurements and eliminate the risk of electric sparks in dusty environments. Compared with the existing sensors, the proposed sensor is intrinsically safer. (2) Using the combination of the FBG sensing capability and the light extinction method, the reflected signal of the FBG offers dual functions: measurement of the dust concentration and measurement of the temperature. The dust concentration and temperature can be simultaneously measured by the central wavelength and the intensity of the reflectance spectrum of the FBG, respectively (3). This research also provides a potential approach for network-based monitoring of distributed dust concentration in large-scale measurements.

## 2. Detection Device and Measuring Principle

**Figure 1:**
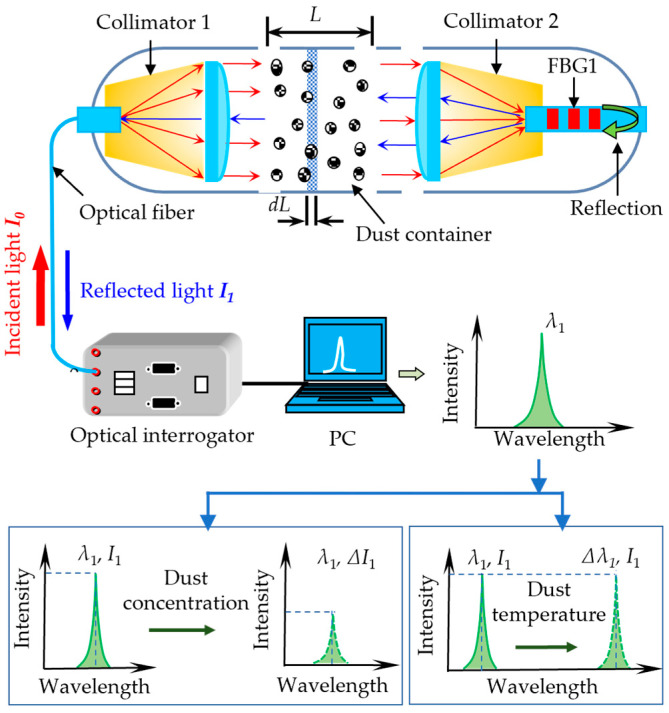
The schematic diagram and working principle of the proposed dust detection system.

As shown in Figure 1, the proposed dust measurement system mainly includes two aligned optical fiber collimators, an FBG, a dust container, a self-developed optical interrogator, and a computer. The fiber pigtail of collimator1 is connected to an optical interrogator via an FC/APC fiber patch cord. The FBG is fabricated in the fiber pigtail of collimator 2 using phase mask technology [23]. The dust container is located between the two collimators.

The internal light source of the optical interrogator generates the incident light with an intensity of *I*_0_, which enters Collimator 1 on the left through a single-mode fiber. The broadband light, after amplification, enters the dust container in the form of parallel beams. The light intensity attenuates due to the reflection and scattering by dust particles. The light then enters Collimator 2 and eventually the FBG on the right side. The part of the light consistent with the central wavelength of the FBG reflects and passes through Collimator 2, the dust container, where the light intensity is again attenuated, and Collimator 1. Finally, the reflected light returns to the optical interrogator. Demodulation yields the real-time intensity *I*_1_ and central wavelength *λ*_1_ of the FBG’s reflection spectrum.

The central wavelength *λ*_1_ and the intensity *I*_1_ of the reflectance spectrum of the FBG are demodulated by a self-developed optical interrogator. The dust concentration and temperature can be simultaneously measured by monitoring the variables *I*_1_ and *λ*_1_, respectively. The detailed measurement principle is given in the forthcoming discussion.

As shown in Figure 1, we divide the space between the two collimators into multiple thin layers of infinitely small thicknesses, each of length *dL*, and the cross-sectional area of each of the thin layers is *S*. Assume that the mesh number of light-absorbing dust particles in each thin layer is *dn*. The total cross-sectional area of all light-absorbing dust particles in the thin layer can be written as follows:

(1) $$dS=\frac{\pi }{4}D^{2}A\left(D,k_{1}\right)dn$$

where *D* represents the particle diameter, *A* is a coefficient related to the gap index *k*_1_, and the particle diameter *D*. After the incident light penetrates the thin layer, we can assume that its intensity attenuation is *dI* and can be expressed in the form of the following equation.

(2) $$-dI=K\left(\begin{array}{ccc} \lambda , & m, & D \end{array}\right)IdS=\frac{\pi }{4}D^{2}K\left(\begin{array}{ccc} \lambda , & m, & D \end{array}\right)A\left(D,k_{1}\right)Idn$$

where *K*(*λ*, *m*, *D*) is the absorption coefficient that is a function of the wavelength *λ* of the input light, the refractive index *m*, and the diameter *D*. The mesh number *dn* contained in the thin layer can be written as the following equation:

*dn* = *k*_3_*N*_v_*S*
*dl* (3)

Combining Equations (1) and (2) yields the equation below.

*dn* = *k*_3_*N*_v_*S*
*dl* (4)

Integrating both sides of Equation (4) gives the following relations:

(5) $$-\int_{I_{0}}^{I_{1}}\frac{dI}{I}=\int_{0}^{I}\frac{\pi }{4}k_{3}D^{2}A(D,k_{1})K(\lambda ,m,D)N_{v}Sdl$$

(6) $$-\ln\frac{I_{1}}{I_{0}}=\frac{\pi }{4}k_{3}D^{2}A\left(D,k_{1}\right)K\left(\begin{array}{ccc} \lambda , & m, & D \end{array}\right)SLN_{v}+Y_{cons}$$

where *Y_cons_* is the constant of integration. Therefore, the relationship between the output light intensity *I*_1_ and dust concentration *N_v_* can be formulated as follows:

(7) $$I_{1}=I_{0}e^{-\frac{\pi k_{3}D^{2}A\left(D,k_{1}\right)K\left(\begin{array}{ccc} \lambda , & m, & D \end{array}\right)}{4}SL\cdot Nv}+Y_{Cons}$$

Thus, after calibration, the dust concentration *N_v_* can be monitored by collecting and analyzing the real-time output intensity *I*_1_ of the reflectance spectrum of the FBG. As per the FBG measurement theory [24], the dust temperature can be measured by detecting the central wavelength *λ*_1_ of the reflectance spectrum of the FBG, as shown in the following equation.

(8) $$\frac{\Delta \lambda }{\lambda }=(\alpha_{f}+\xi )\Delta T+(1-P_{e})\Delta \varepsilon$$

Since no external force acts on the FBG, Δ*ε* = 0 in Equation (8); therefore, the central wavelength variation Δ*λ* is only sensitive to the change in dust temperature Δ*T*.

## 3. Prototype Manufacturing and Experiment

### 3.1. Prototype Manufacturing

**Figure 2:**
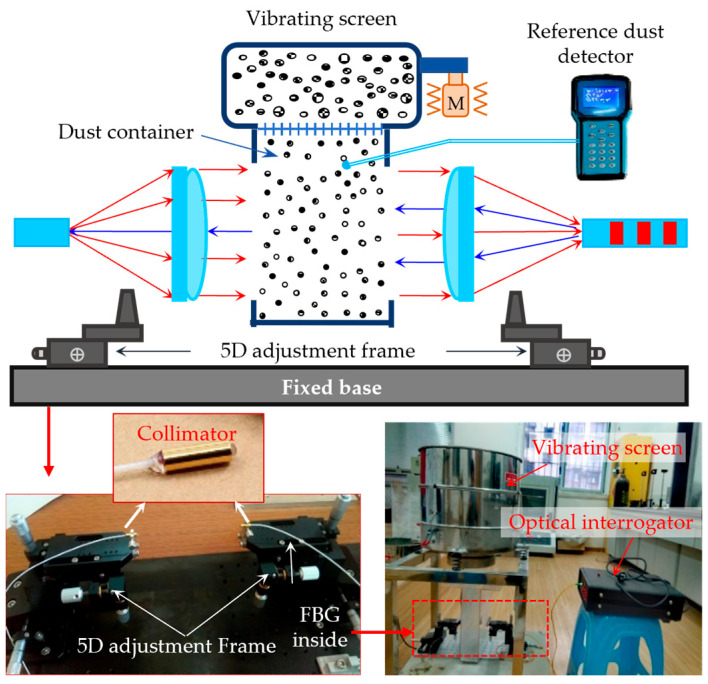
Experimental setup for the dust concentration measurement.

The experimental configuration for the dust concentration test or calibration is depicted in Figure 2. The two fiber collimators are installed on two five-dimensional adjustment frames for easy alignment. The dust container is made of a transparent acrylic board, and two through holes are reserved on both sides of the container to align the collimators. The vibrating screen is placed above the dust container to generate a downward-flowing dust stream. The diameter of the dust particles can be adjusted and controlled by choosing several types of vibrating screens.

**Figure 3:**
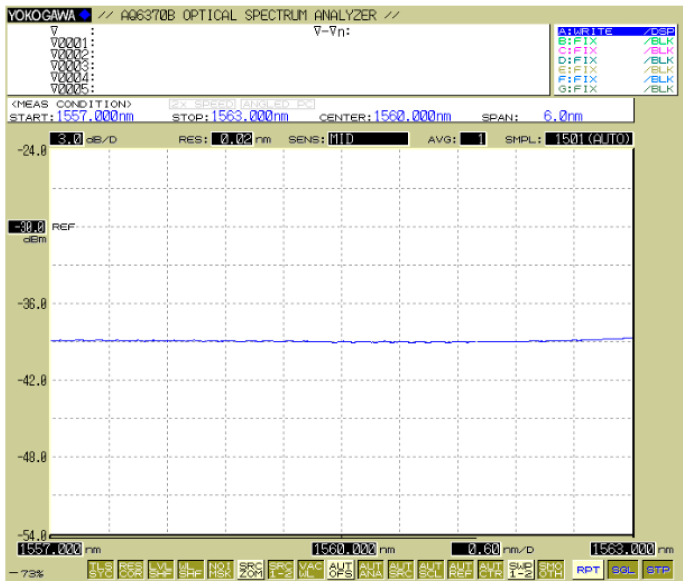
Constant optical power range of the light source.

The self-developed FBG interrogator collects the reflected signals from the FBG (wavelength accuracy: ±3 pm, intensity accuracy: 1 mW, acquisition frequency: 1 Hz). The broadband light source inside the interrogator has a power output of 20 mW and a constant optical power within the 1557.0–1563.0 nm wavelength, as shown in Figure 3, ensuring no change in the optical power with the drift of the FBG wavelength. The original central wavelength of the selected FBG is 1560.1 nm, which lies within the constant optical power range of the light source. During the experiments, a commercial dust detector (EL-1000, Qingdao Yilan Environmental Protection Engineering Co. Ltd., Qingdao, China) with a measurement range of 0–100 g/m^3^ was used as a reference. Moreover, the proposed dust detector comes with a 500 μm spot diameter of the fiber collimators and a working distance range of 10–15 cm.

### 3.2. Evaluation of Measurement Feasibility

As mentioned above, the dust concentration and temperature can be simultaneously measured by monitoring the intensity *I*_1_ and the central wavelength *λ*_1_ of the reflectance spectrum of the FBG. In order to evaluate the measurement feasibility, we have conducted a confirmatory test before calibration.

The test comprised the following two steps. Step 1 involved tapping the vibrating screen laden with dust particles to generate a dust environment exhibiting random concentration fluctuations for approximately 1 min. Meanwhile, the temperature was controlled at around 25 °C during this process. Step 2 consisted of heating the environment using an external heat source in a cyclic “heating–pause” pattern. During this phase, the dust concentration was maintained at zero. A total of four cycles were performed, each lasting approximately 20 s.

**Figure 4:**
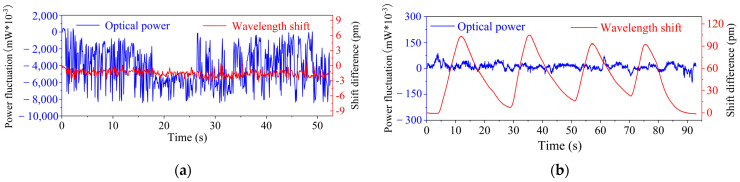
Results of the confirmatory test. (**a**) Test of light wavelength unaffected by dust concentration; (**b**) test of light intensity unaffected by temperature.

The real-time intensity *I*_1_ and the central wavelength *λ*_1_ of the FBG’s reflectance spectrum were both recorded by the optical interrogator throughout testing. Figure 4a and Figure 4b show the time domain responses of the intensity and the central wavelength of the FBG for Step 1 and Step 2, respectively.

From Figure 4a, the intensity curve of the FBG reflectance spectrum oscillates with random changes in dust concentration. On the other hand, the wavelength curve is obviously not sensitive to the change in dust concentration and consistently stable within the range of ±3 pm, corresponding to the accuracy of the interrogator. Conversely, from Figure 4b, the wavelength curve of the FBG shows periodic fluctuations of “rise up and fall down”, corresponding to the cycles of “heating-pause” of the external heating source. Temperature variation does not influence the intensity curve, which is stable within a range of ±0.1 mW.

In summary, the intensity and the central wavelength of the FBG are only sensitive to dust concentration and temperature, respectively. These measurements are decoupled, demonstrating strong agreement with the theoretical principle outlined above and confirming the feasibility of this measurement approach.

### 3.3. Calibration of Dust Concentration and Temperature

The diameter of the dust particle generally depends on the “mesh number,” which refers to the number of holes on the dust screen per square inch. The larger the number, the smaller the diameter of the dust particle. In this research, we selected three different grain sizes of wheat flour to create dust environments, namely 200 meshes (particle diameter of 48~75 μm), 300 meshes (particle diameter of 38~48 μm), and 400 meshes (particle diameter of 25~38 μm). We can obtain different dust concentrations for each dust particle diameter by adjusting the vibrating screen frequency. The calibrated dust concentration is 0–100 g/m^3^.

**Figure 5:**
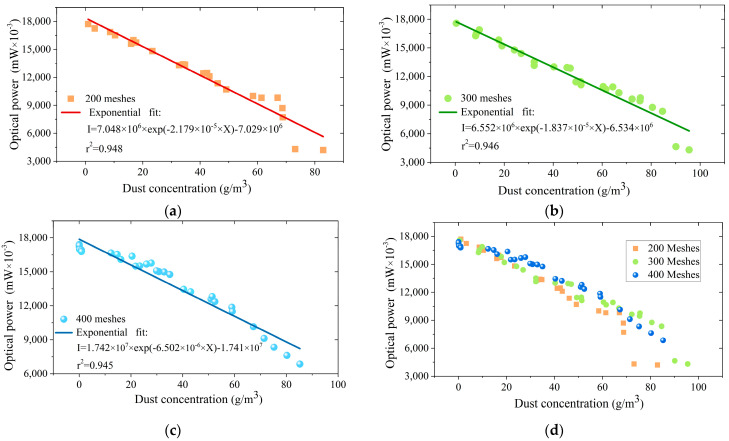
Relationship between the optical intensity of the FBG and dust concentrations of (**a**) 200 meshes, (**b**) 300 meshes, and (**c**) 400 meshes. (**d**) Comparison of test results for different meshes.

The calibration results for the dust concentrations of 200 meshes, 300 meshes, and 400 meshes are shown in Figure 5a, Figure 5b, and Figure 5c, respectively. From Equation (7), we can see that the intensity of the FBG should decrease exponentially with increasing dust concentrations. Therefore, we performed exponential fitting to the calibration data in this experiment. From the calibration results, the FBG intensity change is exponentially related to the dust concentration variation in general. The fitting coefficient *r^2^* for 200 meshes, 300 meshes, and 400 meshes is 0.948, 0.946, and 0.945, respectively.

Figure 5d shows the comparison test results with different meshes. As marked in the red square, when the dust concentration is increased to a certain level, the intensity of the FBG decreases rapidly. The marked data points deviate significantly from the fitted curve. This phenomenon may be due to the adsorption of dust particles because of the large dust concentrations. Abnormally large dust particles may aggregate, potentially causing an optical-path blockage.

**Figure 6:**
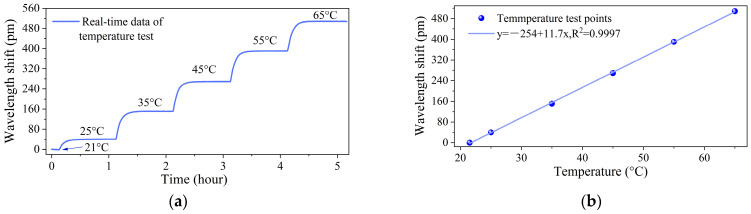
Temperature test results of the FBG with: (**a**) real-time data, (**b**) the relationship between temperature and wavelength shift.

A temperature calibration experiment was conducted within the range of 21~65 °C with increments of 10 °C by an adjustable chamber (GPL-1, Espec Test Equipment Guangdong Co., Ltd., Guangzhou, China; accuracy: ±0.3 °C). As indicated in the dust container, the real-time wavelength–response curves of the FBG perfectly match the temperature variation trend. The relationship between the temperature and the wavelength shift of the FBG is presented in Figure 6b. From the linear-fitted curve, the temperature sensitivity of the sensor is 11.7 pm/°C with *R*^2^ = 0.9997.

### 3.4. Comparison Experiment

To evaluate the accuracy of the proposed detector, a comparative experiment was conducted against the weighing method—the standard reference for an accurate dust concentration measurement. We chose 200-mesh wheat dust as the test object and randomly sampled 18 measurement points with different concentrations. By reading the intensity of the FBG reflected spectrum, combined with the calibration fitting results, we could deduce the concentration measured by the proposed sensor. According to the basic principle of the weighing method, we collected the dust suspended in the dust container and used a high-precision balance to obtain the mass *M*. Knowing that the volume *V* of the dust container is 3.2 × 10^−4^ m^3^, the dust concentration *C* measured by the weighing method can be derived as *C = M/V*.

**Figure 7:**
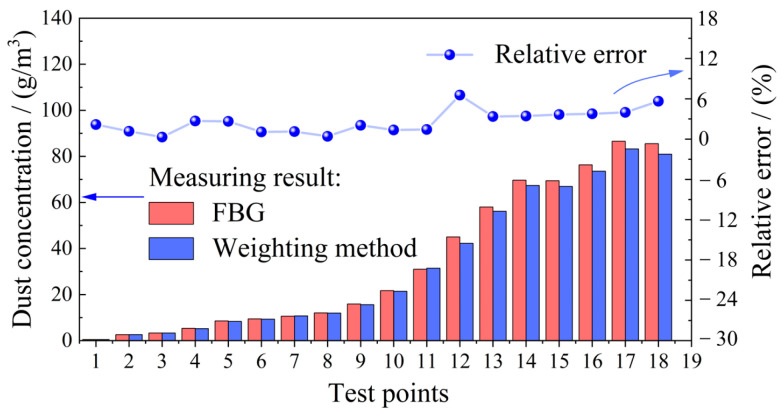
Comparison between the measurement results of the proposed sensor and the weighing method.

The comparison of the measurement results of the proposed detector and the weighing method at each point, along with the relative errors based on the weighing method, is shown in Figure 7. Obviously, when the dust concentration is lower than 30 g/m^3^, the relative error between the results of the proposed detector and the weighing method is mostly within 1.0% and 2.7%. As the dust concentration increases, the relative error also increases gradually. When the dust concentration increases from 45.0 g/m^3^ to 85.5 g/m^3^, the corresponding relative error gradually increases from 3.37% to 6.56%. During this stage, the measurement results of the proposed sensor are generally greater than those of the weighing method.

## 4. Discussion

In this work, we have introduced a hybrid detector for the simultaneous measurement of dust concentration and temperature through the combination of the FBG sensing ability and the optical extinction method. We have objectively summarized the proposed detector’s features and limitations through theoretical analysis, performance evaluation, and comparative testing.

Compared with the existing dust concentration sensors, the proposed sensor is intrinsically safer, which benefits from the inherent characteristics of the FBG sensing mechanism. As the vital sensing components of the proposed detector, the fiber collimator and the FBG are all optical devices requiring no electrical power supply in the dust monitoring site. The proposed detection system only needs a power supply for the optical interrogator in the control room that can be far away from the monitoring site. Only optical signal transmission exists in the actual monitoring site, eliminating the risk of electric sparks caused by electric discharge, short circuit, or circuit failure.

Moreover, the proposed detector has the capability of the simultaneous measurement of dust concentration and temperature. From the evaluation of measurement feasibility, the intensity and the central wavelength of the FBG are sensitive to dust concentration and temperature, respectively, with minute coupling with coupled outputs of ±3 pm and ±0.1 mW. From the calibration experiments, the change in the intensity of the FBG is exponentially related to the variation in the dust concentration. The exponential fitting curves for particles with 200-mesh, 300-mesh and 400-mesh dusts can be described as *I* = 7.048 × 10^6^ × exp (−2.179 × 10^−5^ × X) − 7.029 × 10^6^, *I* = 6.552 × 10^6^ × exp (−1.837 × 10^−5^ × X) − 6.534 × 10^6^ and *I* = 1.742 × 10^7^ × exp (−6.502 × 10^−6^ × X) − 1.741 × 10^7^, respectively. The temperature measurement sensitivity is 11.7 pm/°C.

**Figure 8:**
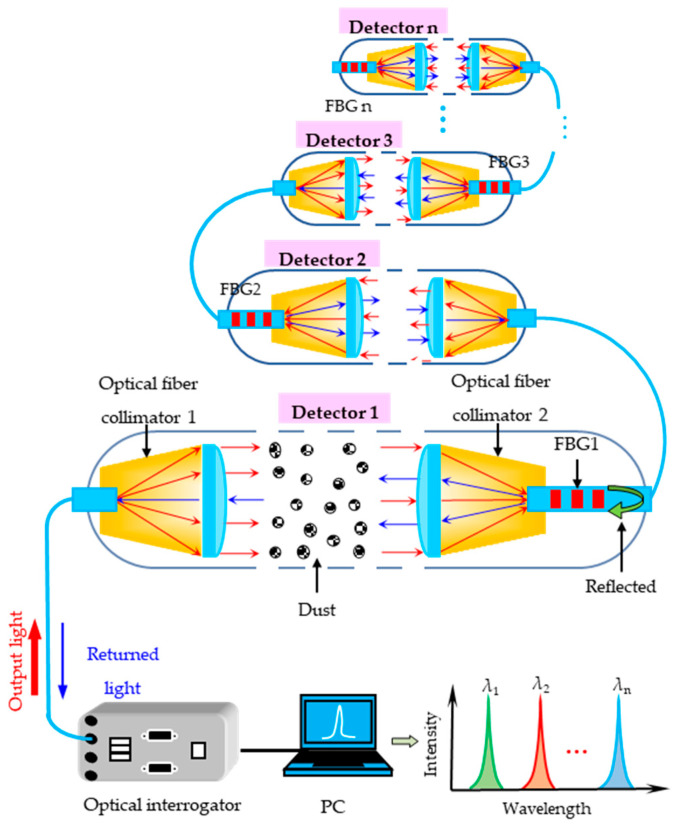
Device and measurement principle of the proposed dust detector in series.

Another potential functionality of the proposed detector is the capability to achieve network-based monitoring of spatially distributed dust concentrations and temperatures. This is because an array of multiple optical detectors along a single fiber can be developed. As shown in Figure 8, multiple detectors connected in series through a single fiber can be distributed at different monitoring points according to actual application requirements. The measured concentration of the nth sensor *C_n_* can be derived as $C_{n}=f\left(\Delta I_{n}-\sum_{i=1}^{i=n-1}I_{i}\right)$, where *I_n_* means the variation in the detected intensity of the nth sensor. Such a configuration supports network-based monitoring in large-scale monitoring applications compared to Ref. [19]. Certainly, in this series arrangement of detectors, factors such as the light source intensity of the demodulator, the sensitivity of photodetection, and the light extinction characteristics of dust will all affect the number of detectors that can be connected in series. For instance, when the light extinction caused by dust is excessive, subsequently connected detectors will receive no light. Additionally, in the design of collimators for detectors, parameters such as the optical path distance between collimators and the spot size of the collimator are critical considerations. These directly determine the dust extinction distance and detectable dust particle size.

As a tentative study, the proposed detector also has its limitations. As presented in Figure 5d, when the calibrated concentration reaches a certain level, the data points deviate significantly from the fitted curve, resulting in low accuracy. Similarly, comparing the proposed sensor and the weighing method shows that the corresponding relative error increases when dust concentration increases. Both these two experimental observations illustrate a phenomenon that dust pollution occurs on the surfaces of the collimators, and there are abnormal blockages of the optical path transmission. Thus, the measured concentration by the proposed sensor will have a larger value than the actual value. It is also consistent with the comparison results that the proposed sensor value for the dust concentration is always larger than the weighing method in Figure 7. In the following research, we will search for a particular film with good light transmission to prevent dust adsorption to avoid polluting the surfaces of the collimators.

## 5. Conclusions

This work proposes and implements a novel hybrid optical fiber detector for the simultaneous measurement of dust concentration and temperature based on the combination of the light extinction method and the FBG sensing approach. The proposed detector is intrinsically safe, eliminating the risks of electric sparks, which may cause dust explosions. Both the theoretical derivation and measurement feasibility evaluation have been performed to investigate the performance.

The experimental calibration results align with the theoretical analysis, demonstrating an exponential relationship between the FBG intensity variation and dust concentration change, thereby validating the theoretical derivation. The temperature sensitivity of the proposed sensor is 11.7 pm/°C. A comparison experiment between the proposed detector and the weighing method demonstrates the variation trend of the relative measurement error with the change in dust concentration. The features and limitations of the detector have been objectively and comprehensively summarized. Future work will involve the elimination of dust pollution on the surfaces of the optical fiber collimators.

## Data Availability

The experimental data are contained within this article.

## Abbreviations

The following abbreviations are used in this manuscript:

FBG	Fiber Bragg grating
LEL	Lower explosive limit
MIE	minimum ignition energy
